# Neuroanatomy of cerebellar mutism syndrome: the role of lesion location

**DOI:** 10.1093/braincomms/fcae197

**Published:** 2024-06-05

**Authors:** Jax Skye, Joel Bruss, Sebastian Toescu, Kristian Aquilina, Amanda Grafft, Gino Bardi Lola, Aaron D Boes

**Affiliations:** Department of Neurology, University of Iowa Carver College of Medicine, University of Iowa, Iowa City, IA 52242, USA; Department of Pediatrics, University of Iowa Carver College of Medicine, University of Iowa, Iowa City, IA 52242, USA; Department of Psychiatry, University of Iowa Carver College of Medicine, University of Iowa, Iowa City, IA 52242, USA; Department of Psychological and Brain Sciences, University of Iowa, Iowa City, IA 52242, USA; Department of Neurology, University of Iowa Carver College of Medicine, University of Iowa, Iowa City, IA 52242, USA; Department of Neurosurgery, Great Ormond Street Hospital, London WC1N 3JH, UK; Developmental Imaging and Biophysics Section, UCL-GOS Institute of Child Health, London WC1N 1EH, UK; Department of Neurosurgery, Great Ormond Street Hospital, London WC1N 3JH, UK; Department of Pediatrics, Division of Developmental and Behavioral Pediatrics, University of Iowa Carver College of Medicine, University of Iowa, Iowa City, IA 52242, USA; Department of Pediatrics, Division of Hematology/Oncology, University of Iowa Carver College of Medicine, University of Iowa, Iowa City, IA 52242, USA; Department of Neurology, University of Iowa Carver College of Medicine, University of Iowa, Iowa City, IA 52242, USA; Department of Pediatrics, University of Iowa Carver College of Medicine, University of Iowa, Iowa City, IA 52242, USA; Department of Psychiatry, University of Iowa Carver College of Medicine, University of Iowa, Iowa City, IA 52242, USA; Iowa Neuroscience Institute, University of Iowa, Iowa City, IA 52242, USA

**Keywords:** posterior fossa tumour, cerebellar mutism syndrome, brainstem tumour, cerebellum disease, cerebellar cognitive affective syndrome

## Abstract

Approximately 25% of paediatric patients who undergo cerebellar tumour resection develop cerebellar mutism syndrome. Our group recently showed that damage to the cerebellar deep nuclei and superior cerebellar peduncles, which we refer to as the cerebellar outflow pathway, is associated with an increased risk of cerebellar mutism syndrome. Here, we tested whether these findings replicate in an independent cohort. We evaluated the relationship between lesion location and the development of cerebellar mutism syndrome in an observational study of 56 paediatric patients ranging from five months to 14 years of age who underwent cerebellar tumour resection. We hypothesized that individuals who developed cerebellar mutism syndrome after surgery, relative to those who did not, would have lesions that preferentially intersect with: (i) the cerebellar outflow pathway and (ii) a previously generated ‘lesion-symptom map’ of cerebellar mutism syndrome. Analyses were conducted in accordance with pre-registered hypotheses and analytic methods (https://osf.io/r8yjv/). We found supporting evidence for both hypotheses. Compared to patients who did not develop cerebellar mutism syndrome, patients with cerebellar mutism syndrome (*n* = 10) had lesions with greater overlap with the cerebellar outflow pathway (Cohen’s d = 0.73, *P* = 0.05), and the cerebellar mutism syndrome lesion-symptom map (Cohen’s d = 1.1, *P* = 0.004). These results strengthen the association of lesion location with the risk of developing cerebellar mutism syndrome and demonstrate generalizability across cohorts. These findings may help to inform the optimal surgical approach to paediatric cerebellar tumours.

## Teaser

Skye *et al*. evaluate the neuroanatomy of cerebellar mutism syndrome in a pre-registered study design. They demonstrate an increased risk of cerebellar mutism when the surgical resection cavity overlaps with (i) the cerebellar outflow pathway and (ii) a previously published lesion-symptom map of cerebellar mutism in paediatric patients, supporting both main hypotheses.

## Graphical Abstract

**Graphical Abstract:**
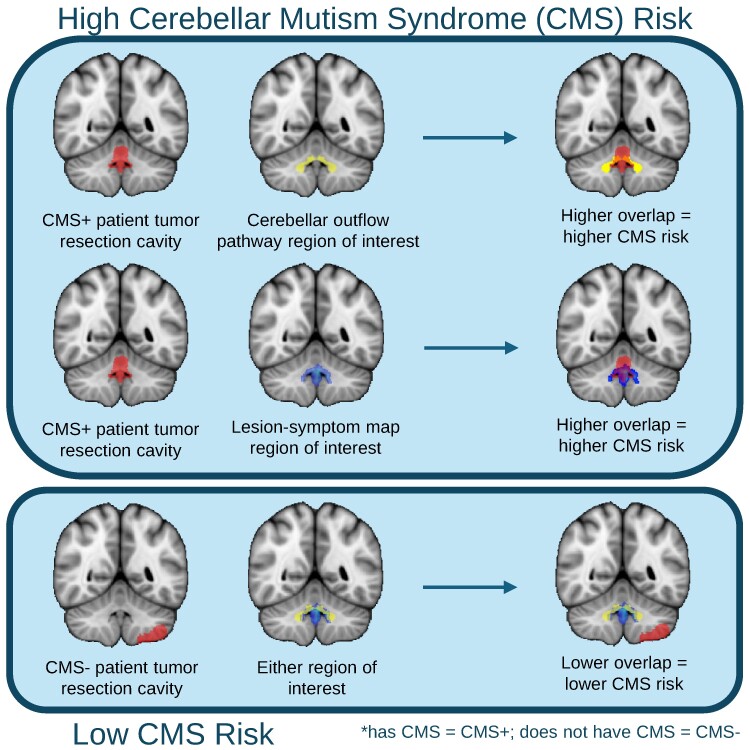

## Introduction

The odds of survival from paediatric brain tumours have steadily improved in recent decades,^1^ escalating the importance of understanding and preventing long-term treatment-related adverse effects. Approximately 25% of children undergoing cerebellar tumour resection will experience a postoperative syndrome characterized by emotional lability, executive dysfunction and language deficits.^2-8^ This constellation of symptoms is commonly referred to as cerebellar mutism syndrome (CMS),^9^ posterior fossa syndrome,^6^ or cerebellar cognitive affective syndrome (CCAS) ± cerebellar mutism^6^; we will use the term CMS for this paper. The duration and severity of this syndrome is variable, but importantly, patients who develop CMS typically have worse long-term cognitive outcomes,^4,6,10-12^ highlighting the importance of this syndrome as a potential harbinger of long-term impairment.

The pathophysiology of CMS is not fully understood. Recent work by our group has demonstrated the importance of lesion location. We hypothesized that damage to the cerebellar outflow pathway, an efferent pathway passing from the deep cerebellar nuclei through the superior cerebellar peduncles to the thalamus, is associated with the development of CMS in paediatric patients.^7^ This hypothesis was formulated in light of the anatomical organization of cerebro-cerebellar communication. The cerebellar outflow pathway is the primary anatomical route that facilitates information flow between the cerebellum and a wide array of brainstem and forebrain regions. The anatomical structure of this pathway acts as a bottleneck, such that relatively small lesions have the potential to disrupt connectivity supporting multiple limbic and cognitive faculties. We found support for this hypothesis from both theory-driven and data-driven analyses.^7^

Identifying lesion sites associated with a higher risk of CMS has the potential to inform clinical practice. It is possible that more personalized prognostic information could be provided to patients and their families about their risk for CMS based on the tumour location relative to the cerebellar outflow pathway. It is also possible that once critical anatomical regions associated with CMS are well established, they could be identified in advance of the tumour resection and used to inform image-guided surgery to avoid those regions when possible. However, it is essential to demonstrate that lesion location is a reliable marker of CMS risk and is generalizable to other independent cohorts if this approach is to be developed for future clinical applications. This was the primary objective of the current study. Specifically, we aimed to evaluate whether the same anatomical regions associated with a higher risk of CMS in our previous study would replicate in an independent cohort. Our anatomical hypotheses and the analytic strategy were pre-registered with the Open Science Framework (https://osf.io/r8yjv/).

In a cohort of 56 patients who underwent cerebellar tumour resection, we hypothesized that patients who developed CMS (CMS+) would have damage to the cerebellar outflow pathway to a greater extent than individuals who did not develop CMS (CMS−). Similarly, we hypothesized that CMS+ individuals would have lesions that overlapped with a lesion-symptom ‘map’ of CMS previously derived from a sample of 195 patients.^7^

## Materials and methods

We analysed clinical outcome and imaging data in patients with cerebellar tumour resection surgery (*N* = 56) from two sites: the University of Iowa (*n* = 9) and Great Ormond Street Children’s Hospital in London (*n* = 47). This study was approved by the Institutional Review Board and ethical standards committee prior to conducting this retrospective study. We included patients under the age of 21 with a diagnosis of a cerebellar tumour that had a surgical resection and follow-up imaging to show the tumour resection cavity. This study focused on a paediatric population since CMS is rare in adult patients.^11^ Each patient also had clinical assessments by their treatment teams to determine whether they met criteria for CMS. CMS was defined by criteria outlined previously: post-surgical onset of reduced speech/mutism and emotional lability.^7^ Emotional lability is a hallmark symptom of CMS.^9^ It was assessed clinically at the bedside by providers involved in post-surgical care. Signs of emotional lability noted in the medical record include irritability, social withdrawal, whining, and crying and laughing inappropriately, all of which caused difficulties in caregiving and in the patient engaging with postoperative physical and occupational therapy. No formal scoring scale was applied in order to grade the severity of the observed behaviour. Additional common features included motor dysfunction or hypotonia. Post-surgical structural neuroimaging scans performed one month or more after the surgery were selected whenever available (see Supplementary Table 1 for exact timing per participant). All scans were reviewed in advance of the analysis and only included if they were of sufficient quality to observe the borders of the post-surgical resection cavity clearly. Scans were performed for clinical indications at each site, so scanners and MRI sequence varied between patients. All MRIs were reviewed and determined to be of sufficient quality to accurately segment the lesion location, which was required for study inclusion. The pons, medulla, brainstem and cerebellum were isolated from the rest of the brain using the ‘isolate’ function in the SUIT toolbox (https://www.diedrichsenlab.org/imaging/suit_function.htm). The same was done for the MNI152 atlas. The highest resolution anatomical sequence (usually T1, MPRAGE) from each scan and the accompanying manually traced lesion mask on the cerebellum and brainstem isolated image was transformed to the brainstem/cerebellum isolated MNI152 1 mm brain atlas using nonlinear transformation with cost-function masking.^13^ The anatomical accuracy of the lesion tracing and the transformation to MNI space was confirmed by a neurologist (A.D.B.) blinded to CMS status.

**Figure 1:**
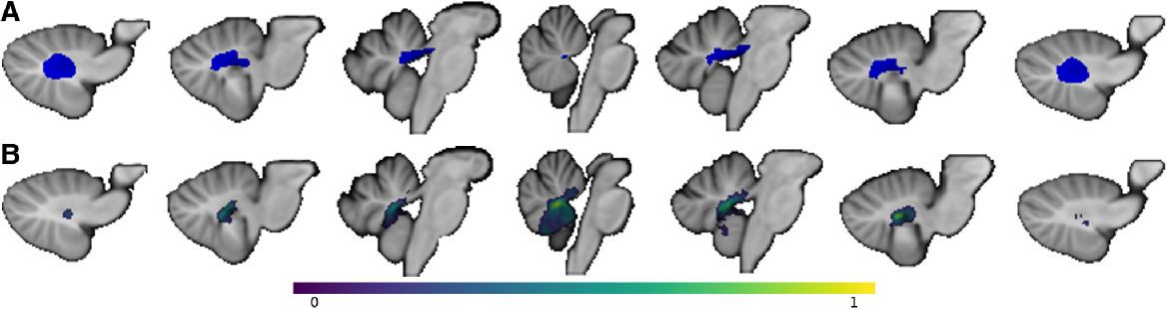
**Cerebellar outflow pathway and lesion-symptom map for cerebellar mutism syndrome (CMS) risk. A** shows the cerebellar outflow pathway. **B** shows the lesion-symptom map from Albazron and colleagues’ 2019 study used to generate the lesion-symptom map lesion load. Voxel values are from 0 to 1 where values closer to 1 represent lesion sites associated with a greater risk for developing cerebellar mutism syndrome (CMS).

Our first hypothesis was that CMS+ patients would have greater disruption to the cerebellar outflow pathway. The region of interest (ROI) used to define the cerebellar outflow pathway is the same as previously described^7^ and is displayed in Fig. 1A. Briefly, it was produced by combining the cerebellar deep nuclei atlas (Spatially Unbiased Infra-Tentorial Template, diedrichsenlab.org/imaging/propatlas.htm) with a mask of the superior cerebellar peduncles defined from a probabilistic atlas of these tracts.^14-16^ The extent to which each individual patient’s lesion location, or lesion ‘mask’, intersected with the binary cerebellar outflow pathway ROI was quantified and referred to as cerebellar outflow pathway ‘lesion load’. The percentage of voxels in each slice of the cerebellar outflow pathway intersecting with the lesion mask was calculated. This required first separating the cerebellar outflow pathway ROI by 1 mm oblique (17°) coronal slices perpendicular to the superior cerebellar peduncles. The slice of the cerebellar outflow pathway with the highest percentage of overlap between the lesion mask and ROI was used as the cerebellar outflow pathway ‘lesion load’ value for that patient. A lesion intersecting all cerebellar outflow voxels in a given slice would have a value of 100% and a lesion that entirely spares this outflow pathway would have a value of 0%. The lesion load values of the CMS+ and CMS− groups were compared using an independent samples *t*-test to evaluate the one-tailed hypothesis that higher cerebellar outflow pathway lesion load will be seen in the CMS+ group compared with the CMS− group.

This study’s second main hypothesis was similar in design to the first but used a data-driven *a priori* ROI in place of the cerebellar outflow pathway ROI. We used the lesion-symptom map generated by Albazron *et al*.^7^ using lesion and outcome data from 195 paediatric patients, displayed in Fig. 1B. The multivariate lesion-symptom mapping was performed using the R package LESYMAP.^17^ LESYMAP uses sparse canonical correlation analysis for neuroimaging (SCCAN) to associate loci of brain damage with CMS status as a binary outcome. A within-sample cross-validation is performed with mapping in 75% of the patients to predict the CMS status of the remaining 25%. This determined the optimal sparseness value with the highest cross-validation correlation between the measured and predicted score.^7,17^ The resulting lesion-symptom map identified cerebellar regions statistically associated with severe postoperative cognitive and affective symptoms, which were referred to as cerebellar cognitive affective syndrome in that study. Each individual in the CCAS group in that study also had mutism or speech deficits and met the criteria for CMS as used in the current analysis. This statistical map showed localization to the cerebellar outflow pathway, specifically the fastigial nuclei, interposed and dentate nuclei, superior cerebellar peduncles and also regions outside of the cerebellar outflow pathway, including lobules IX and X of the vermis.^7^ Using this lesion-symptom map as an *a priori* ROI, we tested our second hypothesis that CMS+ patients will have a higher lesion-symptom map lesion load than patients without CMS. The product of the weighted matrix of voxel values representing the lesion-symptom map, eigenvalue and binary lesion mask of each patient in this study’s cohort is standardized to calculate the lesion-symptom map lesion load. We then compared the lesion-symptom map lesion load between the CMS+ and CMS− groups. A lesion-symptom mapping analysis was also performed in this new cohort (*N* = 56) using the same approach as Albazron and colleagues.^7,17^

### Statistical analyses

One-tailed independent samples *t*-tests were used to compare the cerebellar outflow pathway lesion load and lesion-symptom map lesion load between the CMS+ and CMS− groups. SCCAN was used for lesion-symptom mapping. The alpha level used to determine significance was *P* < 0.05 for all statistical analyses.

### Patient consent

All patients in this study provided informed consent. This study was approved by the ethics review boards of the University of Iowa and Great Ormond Street Children’s Hospital.

## Results

**Figure 2:**
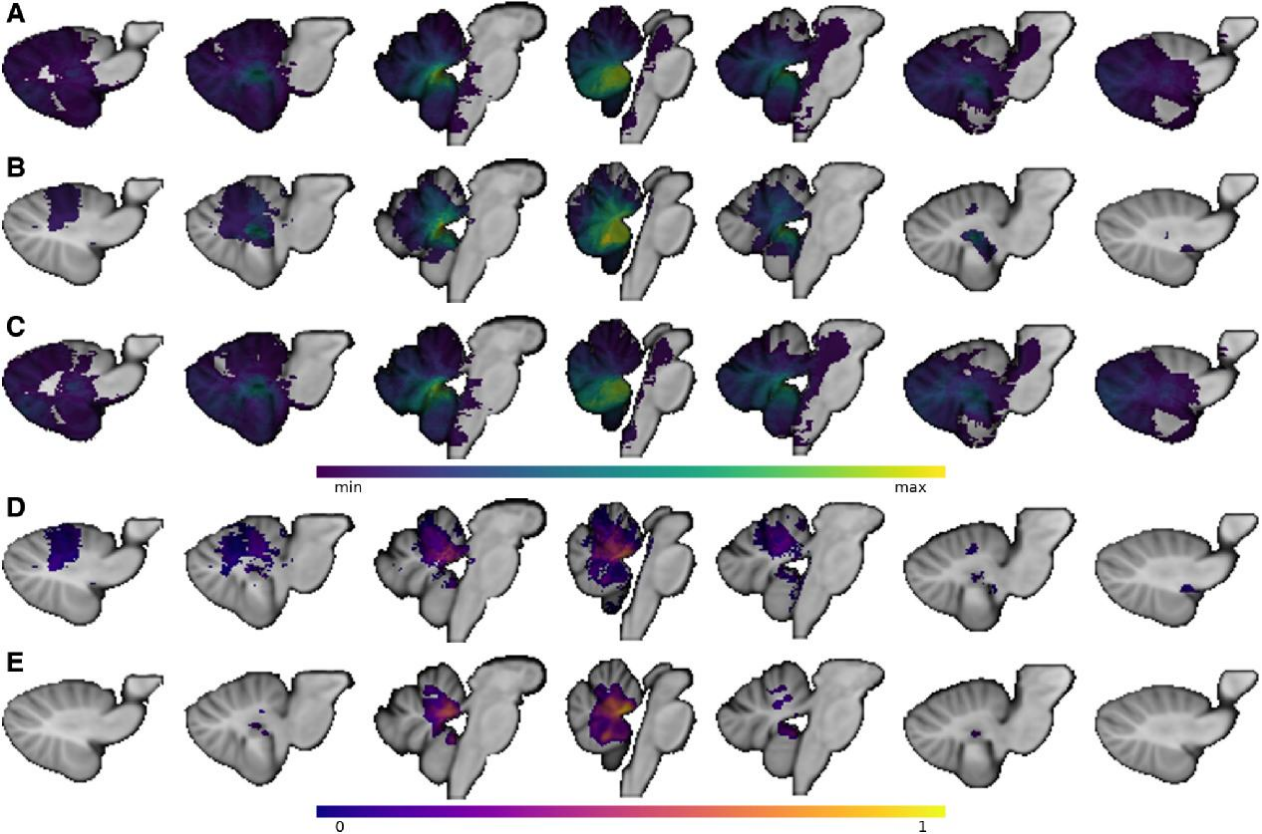
**Neuroanatomy of cerebellar mutism syndrome (CMS).** Voxel values in **A–C** show the number of lesion masks overlapping at that given voxel. **A** shows the lesion overlap of all patients included in this study (*n* = 56). The region of maximum lesion overlap (*n* = 29 of 56) was the right vermian lobule IX (MNI coordinate 4 −53 −34). **B** shows the cerebellar mutism syndrome (CMS+) lesion overlap (*n* = 10) with the maximum overlap at the right vermian lobule IX (9 of 10 lesions, MNI 3 −54 −34). **C** displays the lesion overlap from patients without CMS (CMS−) with peak overlap of 21 of 46 lesions in the right vermian lobule VIII at MNI coordinate 2 −61 −36. **D** shows the proportional subtraction map with a regional peak in the anterior vermis at MNI coordinate 3 −47 −26. The lesion-symptom map in **E** also supports that lesions to the anterior vermis are associated with CMS (*r* = 0.481, *P* < 0.001, peak MNI coordinate 1 −47 −25). Voxel values in **D** and **E** are on an arbitrary continuum from 0 to 1 where values closer to 1 show a stronger association between damage and CMS risk.

A total of 56 paediatric patients (44.6% female) who underwent cerebellar tumour resection met inclusion criteria for the study. Ten patients had CMS (17.9%); of these patients, 30% were female. The average age of the sample is 6.2 ± 3.7 years, median 6.0 years, range five months–14 years with various types of tumours (24 pilocytic astrocytomas, 24 medulloblastomas, 3 atypical teratoid rhabdoid tumours, 2 gangliomas, 1 ependymoma, 1 haemangioblastoma and 1 high-grade glioma; Supplementary Table 1). None of the three patients included in the study under one year of age were noted to have CMS, but they did have other post-surgical symptoms including hypotonia, oropharyngeal dysfunction/dysphagia, cerebellar motor signs, long tract signs and cranial neuropathies. Children with CMS were younger (4.4 years old ± 2.4 years, compared to CMS− 6.9 years old ± 4.2 years; t(21) = −2.59, *P* = 0.016) and had smaller lesion volume that was not significantly different from the CMS group (6076 mm^3^ ± 4891 mm^3^ versus 9831 mm^3^ ± 10 742 mm^3^; t(30) = −1.7, *P* = 0.10; Supplementary Fig. 1). Other variables that were not significantly different between patients from Iowa and Great Ormond Street Children’s Hospital include: age (t(9.53) = 1.93, *P* = 0.0844), lesion volume (t(14.6) = −0.172, *P* = 0.866) and rate of CMS (χ^2^ = 0.450, *P* = 0.503). Medulloblastoma was the tumour type in 70% of CMS+ patients and 32.6% of the CMS− cohort. The lesions of all CMS+ patients crossed the midline, while 65% of CMS− lesions crossed the midline. The lesion masks from each patient were overlapped to show the spatial distribution of the lesions in the entire sample (Fig. 2A) and split by CMS status. CMS+ individuals (*n* = 10) are shown in Fig. 2B and CMS− individuals (*n* = 46) are shown in Fig. 2C. A proportional subtraction map of CMS+ minus CMS− lesion masks is displayed in Fig. 2D.^18^ The lesion-symptom map in Fig. 2E shows that damage in and around the superior cerebellar peduncles and at the roof of the anterior fourth ventricle and vermis is most associated with developing CMS after tumour resection (*r* = 0.481, *P* < 0.001, peak MNI coordinate 1 −47 −25).

**Figure 3:**
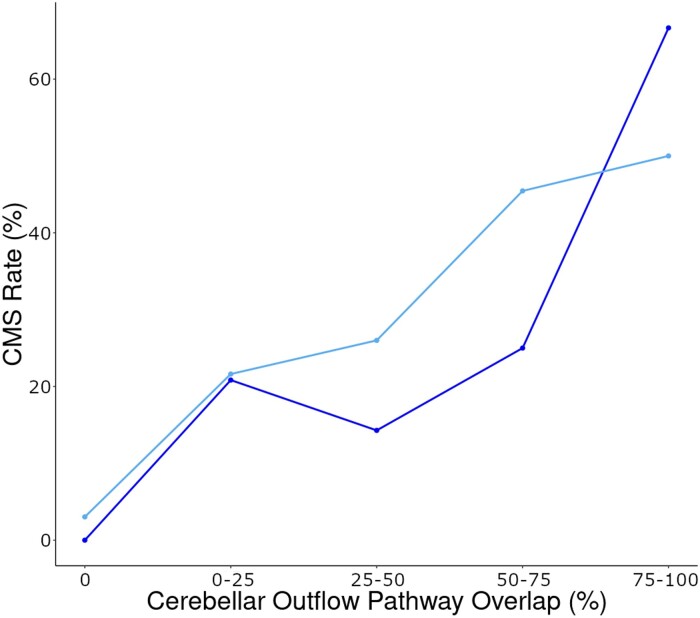
**Cerebellar mutism syndrome (CMS) rate and cerebellar outflow pathway lesion overlap.** The rate of cerebellar mutism syndrome (CMS) development is progressively higher as cerebellar outflow pathway lesion overlap increases. The darker line shows CMS rate in the current study (*N* = 56) and the lighter line shows CMS rate in the Albazron study (*N* = 195).

To test our *a priori* hypothesis that damage to the cerebellar outflow pathway increases the risk of developing CMS, cerebellar outflow pathway lesion load values were calculated for patients with and without CMS. The cerebellar outflow pathway lesion load was higher in the CMS+ group relative to the CMS− group (37 ± 30% versus 19 ± 24%, respectively; t(11) = 1.8, *P* = 0.050; Cohen’s d = 0.73). Notably, the CMS rate was observed to increase in accordance with greater lesion involvement of the cerebellar outflow pathway, replicating a pattern observed previously^7^ (Fig. 3). We evaluated our second hypothesis that CMS+ patients would have a greater lesion-symptom map lesion load when compared to CMS− patients. As hypothesized, CMS+ patients had a greater lesion-symptom map lesion load than CMS− patients (0.018 ± 0.010 versus 0.009 ± 0.011, respectively; t(13) = 3.1, *P* = 0.004; Cohen’s d = 1.11).

## Discussion

This study evaluates lesion location in relation to developing CMS after cerebellar tumour resection in paediatric patients. Our results support our central hypothesis that lesion location influences the risk of CMS.^5-7,19-25^ Specifically, we tested two pre-registered hypotheses and found supporting evidence for both. Individuals who developed CMS had lesions with greater overlap with the cerebellar outflow pathway than those who did not develop CMS. In addition, patients with CMS had lesions that overlapped to a greater extent with a lesion-symptom map of CMS derived from an independent sample.^7^ Surgical resection cavities associated with CMS, as highlighted in the data-driven lesion-symptom mapping analysis, tended to be more anterior and midline and prominently included the superior cerebellar peduncles and parenchyma at the roof of the fourth ventricle in the vermis. Overall, this lesion-symptom map overlapped reasonably well with our prior lesion-symptom map (Fig. 1B versus Fig. 2E) despite the smaller sample size (*N* = 56 versus 195). Our result also supports the higher incidence of CMS with lesions that cross the midline and with medulloblastoma relative to other tumour types.

Studies over the past few decades have emphasized the role of the cerebellum in an array of cognitive domains including working memory, language and socioemotional function.^26^ Studies of CMS help to further clarify the important role of the cerebellum in the development of these cognitive and socioemotional functions, along with identifying which specific regions are most critical.^7,19^ The association of vermian lobule IX lesions and CMS is consistent with a role for this cerebellar region in both motor and non-motor functions. Non-motor functions are thought to include cognition, autonomic function, limbic, emotional and social behaviour (reviewed in Manto *et al*.^27^).

The fastigial, interposed and dentate nuclei comprise the deep cerebellar nuclei, which together provide the major source of output from the cerebellum. A pathway from the deep nuclei through the superior cerebellar peduncles to the brainstem, thalamus and cerebral cortex forms a loop that then projects back to the cerebellum via cerebro-pontine-cerebellar pathways.^28^ This reciprocal loop carries information relevant for several adaptive functions that span executive function, language processing and visuospatial cognition amongst other cognitive and socioemotional abilities.^26^ The integrity of this anatomical loop influences behaviour across different time scales as it appears to be important for ongoing behaviours and the typical acquisition of cognitive and socioemotional ability over the course of development. Thus, damage to the cerebellar outflow pathway has the potential to impact a wide variety of functions seen with CMS due to it being an anatomical bottleneck in the information flow between the cerebellum and the rest of the brain, with both immediate and long-term consequences. This matches the time course of deficits in CMS that are observed acutely but also have been associated with long-term impairments. This cerebro-cerebellar disconnection leads to diaschisis,^29^ the interruption of a pattern of brain activity in regions spatially distant from the site of a lesion. It also leads to a developmental diaschisis, with areas of the brain disconnected from the cerebellum developing differently than they would have with an intact cerebro-cerebellar connection.^30,31^ Thus, damage to the cerebellar outflow pathway can have far-reaching consequences on activity in regions more canonically associated with cognitive and affective function in both acute and chronic epochs.

Taken together, the current findings emphasize that lesion location is useful in predicting the development of CMS in a way that generalizes across cohorts. We hope that this line of research evaluating lesion anatomy and other risks for developing CMS will ultimately help to inform ongoing efforts to reduce the likelihood that a patient will develop CMS post-surgery. What are the future directions for these efforts? First, it would be valuable to continue this line of research in much larger multi-site cohorts made possible through large-scale collaboratives such as the Children’s Oncology Group. This would allow for more sophisticated models that include lesion anatomy with other risk factors to maximize the amount of variance in CMS outcome that can be explained. Further, this work was conducted on post-surgical scans. It may be possible to transform the critical anatomical regions identified from post-surgery studies conducted on a template brain (e.g. MNI) onto the pre-surgical anatomical images of individual patients, such that prospective clinical trials could be designed to investigate MRI-guided surgical interventions that provide optimal information about the anatomy of critical CMS regions to the surgeon during the operation. Further, it may be possible to provide patients and their families with more accurate prognostic information about both CMS risk and long-term cognitive risk based on tumour and resection cavity location as we gain further knowledge of these relationships in larger studies.

There are limitations to this study. First, CMS was diagnosed by the treating clinicians without standardized assessments of behavioural deficits. New assessment tools that are now available may be useful for future studies.^32^ It is possible that symptom-specific quantitative assessments may further clarify unique anatomical associations with specific symptoms. In addition, the lesion anatomy was extracted from clinical imaging, which sometimes lacks the high resolution of research-quality MRI. Other factors that likely influence the development of CMS, like post-surgical treatment plan, surgical approach, oedema, hydrocephalus and premorbid cognitive abilities, were not evaluated here. Similarly, dysphagia is a common symptom associated with CMS that was not systematically assessed in our patients. The observation that lesion location significantly relates to CMS outcome without accounting for these other variables supports the robust effect of lesion location. Still, more sophisticated models that take these additional factors into account are likely to explain additional variance regarding CMS risk.

## Conclusion

In closing, this study provides additional evidence that damage to critical regions of the cerebellum and its outflow pathway is associated with an increased risk of a child developing CMS after cerebellar tumour resection. Further work in this line of research could be used to inform the surgical approach to paediatric cerebellar tumour resection. For instance, critical anatomical regions that, when resected, are associated with increased CMS risk could be displayed as an overlay onto a patient’s preoperative MRI scan so that surgeons could design a minimally invasive MRI-guided approach that minimizes damage to these regions.

## Supplementary Material

fcae197_Supplementary_Data

## Data Availability

Data from this study are posted on Open Science Forum. This includes spreadsheets with demographic data, CMS status, lesion masks, regions of interest and lesion load values, along with scripts (https://osf.io/9gqbu/?view_only=e6a83d1168754dd3927d75fea28db71b).
