# Supplementary material for: Neuroanatomy of cerebellar mutism syndrome: the role of lesion location
Source: Brain Commun. 2024 Jun 5;6(4):fcae197. doi: 10.1093/braincomms/fcae197 (PMC11250198; doi:10.1093/braincomms/fcae197)
Supplement: fcae197_Supplementary_Data [file fcae197_supplementary_data.docx]

| **Surgery – Scan Latency (days)** | **Age at Surgery (years)** | **Tumor Type** | **CMS Status** | **Cohort** | **Surgery – Scan Latency (days)** | **Age at Surgery (years)** | **Tumor Type** | **CMS Status** | **Cohort** |
| --- | --- | --- | --- | --- | --- | --- | --- | --- | --- |
| 136 | 5.13 | PA | Yes | GOSH | 100 | 3.06 | PA | No | GOSH |
| 102 | 2.15 | MB | Yes | GOSH | 98 | 7.20 | PA | No | GOSH |
| 125 | 4.07 | MB | Yes | GOSH | 97 | 6.06 | MB | No | GOSH |
| 234 | 2.30 | MB | Yes | GOSH | 128 | 11.92 | PA | No | GOSH |
| 62 | 1.42 | ATRT | Yes | GOSH | 64 | 4.33 | MB | No | GOSH |
| 105 | 6.70 | MB | Yes | GOSH | 101 | 0.44 | ATRT | No | GOSH |
| 106 | 5.92 | MB | Yes | GOSH | 138 | 2.31 | PA | No | GOSH |
| 83 | 4.36 | PA | Yes | GOSH | 101 | 5.79 | PA | No | GOSH |
| 330 | 9.91 | MB | Yes | Iowa | 100 | 5.44 | PA | No | GOSH |
| 19 | 3.19 | MB | Yes | Iowa | 92 | 9.84 | PA | No | GOSH |
| 103 | 6.72 | PA | No | GOSH | 101 | 9.54 | PA | No | GOSH |
| 109 | 8.66 | PA | No | GOSH | 123 | 9.12 | MB | No | GOSH |
| 90 | 1.30 | GG | No | GOSH | 93 | 11.50 | PA | No | GOSH |
| 116 | 7.38 | MB | No | GOSH | 108 | 2.47 | PA | No | GOSH |
| 99 | 9.29 | PA | No | GOSH | 98 | 3.90 | PA | No | GOSH |
| 126 | 11.18 | PA | No | GOSH | 114 | 3.65 | MB | No | GOSH |
| 251 | 2.45 | EP | No | GOSH | 97 | 8.62 | PA | No | GOSH |
| 176 | 0.45 | MB | No | GOSH | 112 | 4.46 | MB | No | GOSH |
| 191 | 0.66 | ATRT | No | GOSH | 85 | 2.46 | MB | No | GOSH |
| 134 | 7.50 | PA | No | GOSH | 103 | 8.00 | MB | No | GOSH |
| 90 | 1.13 | PA | No | GOSH | 132 | 3.52 | MB | No | GOSH |
| 79 | 12.30 | HGG | No | GOSH | 7 | 8.07 | MB | No | Iowa |
| 222 | 4.61 | PA | No | GOSH | 2 | 14.01 | MB | No | Iowa |
| 149 | 8.49 | GG | No | GOSH | 5 | 13.01 | MB | No | Iowa |
| 102 | 6.80 | PA | No | GOSH | 99 | 2.30 | MB | No | Iowa |
| 77 | 14.56 | HAB | No | GOSH | 218 | 7.73 | MB | No | Iowa |
| 109 | 9.66 | PA | No | GOSH | 22 | 8.01 | MB | No | Iowa |
| 79 | 10.73 | PA | No | GOSH | 108 | 18.67 | MB | No | Iowa |
| *GOSH – Great Ormond Street Children’s Hospital, PA – pilocytic astrocytoma, MB – medulloblastoma, GG – ganglioglioma, ATRT – atypical teratoid rhabdoid tumor, EP – ependymoma, HGG – high-grade glioma, HAB – hemangioblastoma | | | | | | | | | |

**Supplementary Table 1** Raw data for each patient is shown. “GOSH” indicates patients from Great Ormond Street Children’s Hospital and “Iowa” indicates patients from the University of Iowa.


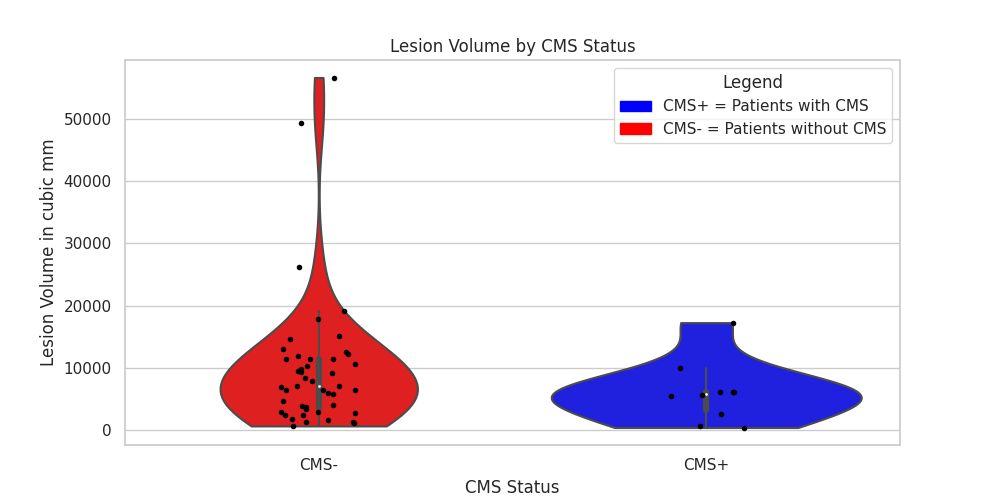


**Supplementary Figure 1** Lesion volume in mm^3^ is shown for each patient with cerebellar mutism syndrome (CMS+) and without CMS (CMS-). CMS+ patients had a smaller lesion volume on average (6076 mm^3^ ± 4891 mm^3^) compared to CMS- patients (9831 mm^3^ ± 10742 mm^3^), but this difference was not significant (t(30)=-1.7, p=.10).
